# Mechanical properties of rock under uniaxial compression tests of different control modes and loading rates

**DOI:** 10.1038/s41598-024-52631-1

**Published:** 2024-01-25

**Authors:** Zhilei He, Guoli Wu, Jun Zhu

**Affiliations:** 1 Research Institute of Geotechnical Engineering and Hydraulic Structure, North China University of Water Resources and Electric Power, Zhengzhou, 450046 China; 2 Zhongshui Zhujiang Planning Survey and Design Co., Ltd., Guangzhou, 510610 China

**Keywords:** Civil engineering, Mechanical engineering

## Abstract

To study the influence of control mode and loading rate on mechanical property of rock, uniaxial compression tests of four types of rocks (gray sandstone, red sandstone, mudstone and granite) are carried out under axial strain control mode and lateral strain control mode respectively. The characteristics of complete stress and strain curves, strength, brittleness and failure modes are analyzed. The results show that control mode has little influence on the pre-peak deformation, stress thresholds, while it has a greater impact on post-peak stress and strain curve, which makes the post-peak deformation stable and controllable, and shows the feature of Class II behavior. With lateral loading rates decrease, post-peak stress and strain curves appear more and more obvious fluctuations in the post-peak stage, and the time required for rock failure increases sharply, but the lateral control rate has little effect on the brittleness of rock. The failure mode of rock samples under axial strain control mode is mainly splitting failure, while that under lateral strain control is gradually changed to shear failure. The smaller the lateral loading control rate is, the more obvious the characteristics of shear failure is. Compared with uniaxial compression tests, under high confining pressure, the lateral dilation deformation is restricted, so peak strength is larger and stress redistribution can be better adjusted and stress fluctuation reduced accordingly in post-peak stage. The research results are an effective supplement to rate-dependent property of rocks and can provide some reference for deformation and strength characteristics research of brittle rock under lateral control mode.

## Introduction

The complete stress and strain curve of rock has been recognized to be a useful indicator to interpret the strength and deformability of rock in uniaxial compression tests. Great effort has been made to experimentally obtain the complete stress and strain curve (CSSC) of rock sample. The complete stress and strain curve of rock are not only closely related to rock type and lithology, but also related to the control mode and control rate or loading rate used in the test.

It is well-known that there are three common control modes (axial load control mode, axial strain control mode and circumferential or lateral strain control mode) in indoor rock mechanics experiment. Axial strain control mode is the most commonly used. In axial strain control mode, rock samples are applied displacement at a certain rate in axial direction until the rock samples failure. The complete stress–strain curve can be obtained for soft and moderately brittle rocks. However, for high brittle rock, once rock enters into the post-peak deformation stage, there is a sudden drop of axial stress and result in catastrophic failure. The complete stress–strain curve can’t be obtained. To overcome this problem, some modified test control modes or methods were proposed to slow down the rock damage at the post-peak stage, such as axial + lateral displacement control mode^1^, stress–strain linear combination control mode^2,3^, stress return servo control mode^4^ and so on. ISRM suggested methods of obtaining the complete stress–strain curve is the circumferential stain control mode for the high brittle rock^5^. Compared with axial strain control, circumferential or lateral strain control mode is that lateral strain can be monotonically increased by the program signal without leading to an unstable failure of the specimen. The rock sample is controlled to dilate uniformly. With sufficiently slow ramp rates and enough resolution in the program signal source of servo control system, lateral control will permit post-failure testing of the most difficult rock sample. According to the characteristic of complete stress and strain curve, Wawersik and Fairhurst^6^ divided the post-peak curves of CSSC into Class I and Class II types. Table 1 lists the result of recent years important rock compression tests using lateral strain control mode from the literature. It is seen that lateral strain control usually lead to Class II or mixed Class I and Class II curve and axial stain control usually lead to Class I type curve.

**Table 1:** Rock compression tests using the lateral strain control from the literatures.

Materials	Test type	Control mode	Strain rate/displacement rate	Class I or Class II	References
Zigong sandstone	Uniaxial	Lateral strain	2 × 10^–6^/s	II	Zhang et al.^7^
Granite and marble	Uniaxial and triaxial	Axial stain	15 × 10^–6^/s	I	Zhang and Li^8^
		Lateral strain	4.2 × 10^–6^/s	II	
Granite	Uniaxial and triaxial	Axial stain	0.001 mm/s	I	Wong^9^
		Lateral strain	0.00067 mm/s	II Mixed I and II	
Marble, sandstone, granite and basalt	Uniaxial and triaxial	Axial stain	1 × 10^–6^/s	I	Hou^10^
		Lateral strain	1 × 10^–5^/s	II Mixed I and II	
Mortar	Uniaxial	Axial stain	10^–6^/s	I	Wang^11^
		Lateral strain	10^–6^/s; 2 × 10^–6^/s; 3 × 10^–6^/s; 2 × 10^–7^/s	II I and II	
Coal	Uniaxial and triaxial	Axial stain	0.001 mm/s	I	Tang^12^
		Lateral strain	0.001 mm/s	II Mixed I and II	
Kuru granite	Uniaxial	Lateral strain	250 µε/min	II	Wan^13^

Except for the control mode, control rate or loading rate also influence the shape of the complete stress and strain curve. In other words, the rock materials have a property of rate dependent and loading rate influences the mechanical property of rock samples. The loading rate is a parameter with a wide range of value, so we only discuss the issue in the quasi-static strain rate (10^–5^–10^–1^ s^−1^) here, which is the rigid servo test machine can operate well. Loading rate has an important effect on strength and deformation property of rock samples. On the one hand, initial cracking stress and peak strength increase with the loading rate. But the different rocks have different sensitivity to loading rate. In elastic stage, the loading rate has little effect on the mechanical properties. In crack propagation stage, the loading rate has significant influence. da Huang et al.^14^ studied the correlations between initial cracking stress, peak strength and loading rate based on the uniaxial compression tests of coarse crystal grain marble with nine strain rate levels. Chengdong et al.^15^ performed a series of uniaxial compression tests at 6 strain rates in the range of 2 × 10^–5^ to 5 × 10^–3^ s^−1^. They found that the peak strength was positively correlated with the axial loading rate. Yanwei et al.^16^ studied axial loading rate effect of coal rock, and found that the strength of coal increased with the increase of the loading rate, but it is not sustainable. When rising to a certain extent, the strength no longer increases, but began to decrease. On the other hand, the elastic modulus and deformation modulus have a closely related to the loading rate. In general, the elastic modulus and deformation increase with the loading rate. Weiguo et al.^17^ found the elastic modulus of salt rock increase with loading rate, but by a smaller margin. Poisson’s ratios decrease with loading rate increase. Zhang et al.^18^ studied the axial loading rate effect on the mechanical properties of deep sandstones, and found the elastic modulus increase with the loading rate and the failure mode transforms form plastic–elastic–plastic to plastic–elastic with the loading rate. Besides, Xiaotao et al. ^19^ found the loading rate enhances the mechanical and deformation features of rock material at meso-scale, which makes the rock breaking into pieces and causes energy loss increasingly by particle flow code.

Although the above results state the control mode and loading rate has an important effect on the mechanics properties of rock samples, there are still some problems worthy of further study. For example, the effect of the loading rate on the rock mechanical characteristics is mainly studied under the axial control mode, whether lateral strain rate also influences the mechanics and deformation property of rock in lateral strain control mode or not, what is the different of loading rate under axial strain control and lateral strain control? Are there same mechanical response for hard and brittle and soft rock? In view of this, a series of uniaxial compression tests of four types rock (gray sandstone, red sandstone, mudstone and granite) were carried out under axial strain control and lateral strain control mode respectively. By changing the pre-defined lateral strain control rate, the influence of lateral loading rate on rock strength and deformation characteristics is studied, and the characteristics of rock stress and strain curve and failure mode under different control rates are analyzed. The rate effect of lateral strain control mode is an effective supplement to the rock rate effect problem, and provides a reference for obtaining the complete post-peak stress and strain curve of brittle rock.

## Experimental set-up

### Experimental equipment

**Figure 1:**
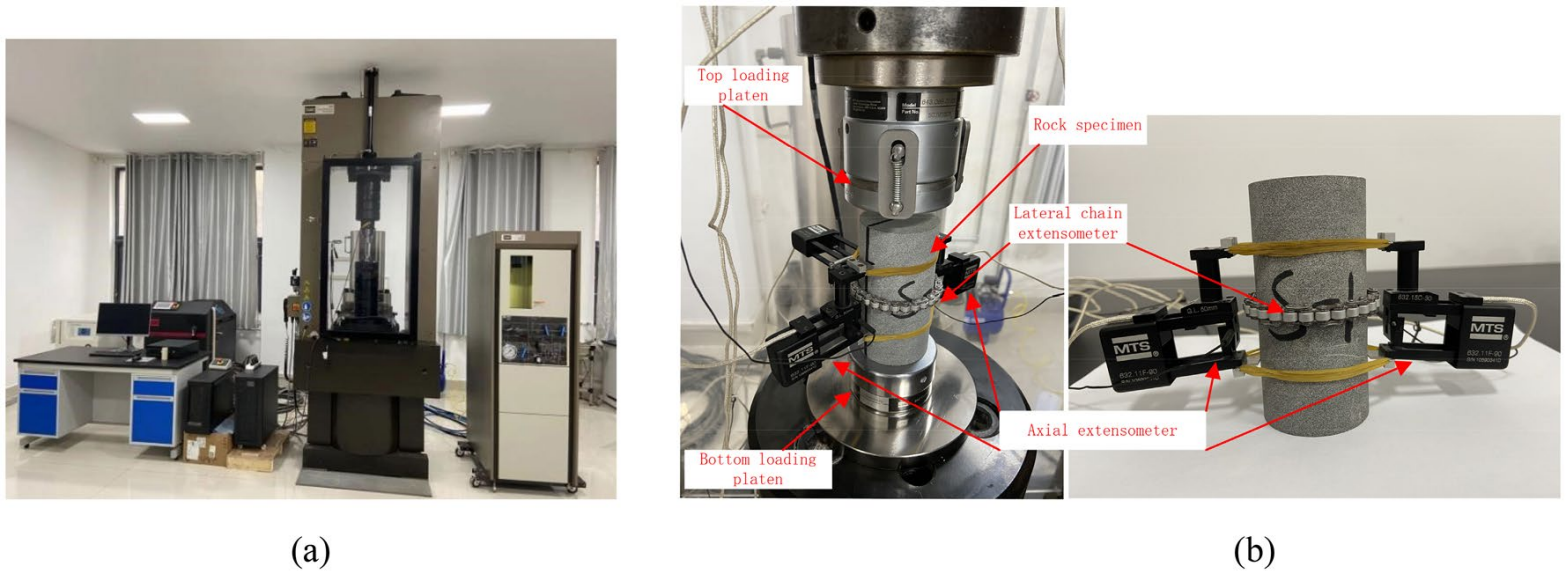
MTS815 rock mechanics test machine.

Rock uniaxial compression tests are carried out on the MTS815 closed-loop servo-controlled hydraulic compressive machine. The experimental equipment is shown in Fig. 1a. The MTS 815 test machine can provide a maximum pressure of 4600 kN, a maximum tension of 2300kN, a maximum confining pressure of 140MPa, a frame stiffness of 10.5 × 10^9^ N/m. The equipment often can be used to measure the deformation and strength parameters of rock and concrete. Figure 1b shows the sample installation and strain gauge. Two axial extensometers are attached on the sample middle half-zone to measure axial strains on opposite sides of the specimen, the gage length of the two axial extensometers is 50 mm, so axial deformation of the 50 mm range can be precisely recorded. The lateral chain extensometer are designed to accurately measure circumferential strains on specimens.

The axial strain also can be measured by the platen displacement from the actuator or external linear variable differential transducer (LVDT). The axial extensometer can measure the true deformation for attaching on the surface of the specimen, but the range of gage length constraints the measured strain value. In post-peak stage, the local fracture of the surface of rock samples may result in that data of the axial extensometer may be not completely consistent, even be invalid^9,20^. The platen displacement includes the whole deformation of the full length of rock, and does not affected by local fracture of rock. The measure data is more stable. Therefore, the axial strain from the platen displacement is adopted to plot the complete stress and strain curves.

### Sample preparation and loading scheme

Four types of rock, gray sandstone, red sandstone, mudstone and granite were prepared with a required dimension of 50 mm in diameter and 100 mm in height following the ISRM suggested method. The rock specimens containing no fissures with good homogeneity were selected to carry out the compression experiments. Axial strain control mode and lateral strain control mode were adopted in tests. Axial strain control mode is that axial force are applied at a per-defined rate, such as 0.001 mm/s or 0.002 mm/s, and keep the loading rate until the rock sample failure. While lateral strain control mode is actually two-stage loading. In the first stage, the axial force is applied at a per-defined strain or displacement rate, and until the axial force reach to a certain value (50% of the peak strength), and then control mode will be switched from the axial strain to lateral strain, and then axial force will continually applied by the program signal from servo system at the pre-defined lateral loading rate.

Control mode and loading rate for four types rock samples are listed in Table 2. For gray sandstone, uniaxial compression tests are conducted. Under axial strain control mode, the axial strain loading rate is 0.001 mm/s. Under lateral strain control mode, the axial strain loading rate at first stage is 0.001 mm/s, and the lateral loading rate at second stage is 0.0001, 0.0003, 0.0005 mm/s respectively. For red sandstone, uniaxial compression tests are also conducted. Under axial strain control mode, the axial strain loading rate is 0.001 mm/s and 0.002 mm/s respectively. Under lateral strain control mode, the axial strain loading rate at first stage is 0.001 mm/s, and the lateral loading rate at second stage is 0.001 mm/s. For mudstone, uniaxial compression tests are also conducted. Under axial strain control mode, the axial strain loading rate is 0.002 mm/s. Under lateral strain control mode, the axial strain loading rate at first stage is 0.002 mm/s, and the lateral loading rate at second stage is 0.005 mm/s. For granite, uniaxial compression tests and triaxial compressions are conducted. Granite is hard and brittle and easy to be severely damaged and violence failure without confining pressure, so only lateral strain control mode are carried out in uniaxial compression tests. Under lateral strain control mode, the axial strain loading rate at first stage is 0.002 mm/s, and the lateral loading rate at second stage is 0.005 mm/s and 0.0005 mm/s respectively. The confining pressure is 40 MPa in triaxial compression tests, the axial strain loading rate is 0.004 mm/s under axial strain control mode, and the axial strain loading rate at first stage is 0.004 mm/s, and the lateral loading rate at second stage is 0.005 mm/s under lateral strain control mode.

**Table 2:** Control mode and loading rate for four types rock.

Rock type	Control mode of the first stage	Loading rate (mm/s)	Control mode of the second stage	Loading rate (mm/s)	Confining pressure
Gray sandstone	Axial strain	0.001	–	–	–
	Axial strain	0.001	Lateral strain	0.0001	–
	Axial strain	0.001	Lateral strain	0.0003	–
	Axial strain	0.001	Lateral strain	0.0005	–
Red sandstone	Axial strain	0.001	–	–	–
	Axial strain	0.002	–	–	–
	Axial strain	0.001	Lateral strain	0.001	–
Mudstone	Axial strain	0.002	–	–	–
	Axial strain	0.002	Lateral strain	0.005	–
Granite	Axial strain	0.002	Lateral strain	0.005	–
	Axial strain	0.002	Lateral strain	0.0005	–
	Axial strain	0.004	–	–	40 MPa
	Axial strain	0.004	Lateral strain	0.005	40 MPa

## Test results and analysis

### Characteristics of complete stress and strain curve

**Figure 2:**
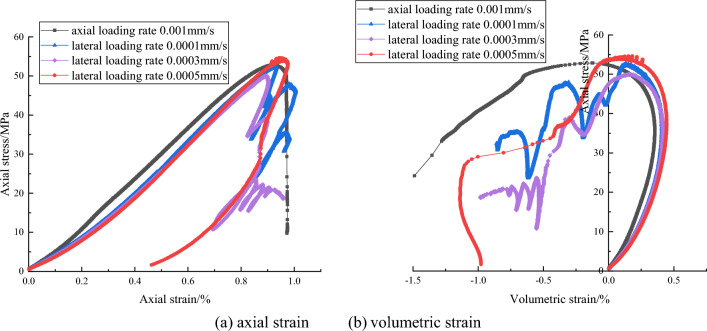
The complete stress and strain curve of gray sandstone under different lateral strain rate and control mode.

The complete stress and strain curves of gray sandstone are shown in Fig. 2. The complete stress and strain curves include a pre-peak strain hardening stage and a post-peak damage softening stage. The pre-peak stress and strain curves almost overlap very well under different lateral loading rates, axial stress increases as the axial strain increases until the peak strength. At the peak point, the peak strength is around 52 MPa and the peak strain is around 0.009, indicating that the effect of lateral loading rate on the pre-peak deformation, peak strength and peak strain is basically the same. However, in the post-peak deformation stage, axial stress and axial strain decrease at the same time, showing the characteristics of the Class II behavior. What’s more, with lateral loading rates decrease, stress and strain curves appear more and more obvious fluctuations in the post-peak stage. Axial stress appear repeatedly cycle of falling and rising phenomenon. The volumetric strain-axial stress curve and axial strain–axial stress curves show similar behaviors. Compared with the responses under the lateral strain control, the pre-peak deformation is similar but post-peak behavior is different under axial strain control mode. The axial stress decreases and axial strain is increases, showing the characteristic of Class I behavior in the post-peak stage.

**Figure 3:**
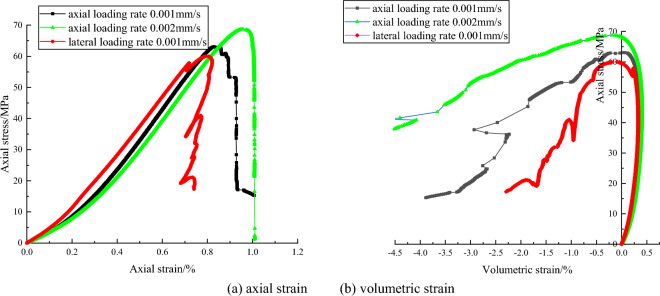
The complete stress and strain curve of red sandstone under different loading rate and control mode.

The complete stress and strain curves of red sandstone are shown in Fig. 3. From the Fig. 3, under axial strain control mode, the variation trends of the stress and strain curves are similar. In compaction stage at the beginning of loading, the axial stress increases slowly, but the stress increases almost linearly to the peak strength in subsequent elastic stage and then decreases abruptly to a low value in post-peak stage. With the axial loading rate increase, the peak strength also increase from 63 to 69 MPa. The faster the axial loading rate is, the greater the peak strength of red sandstone is. Under lateral strain control mode, the characteristic of complete stress and strain in the pre-peak stage is also similar to the same loading rate under axial strain control mode. Peak strength of them also approximately equal. However, characteristic of post-peak is different. The axial stress decreases and axial strain also decreases, showing the feature of Class II behavior under lateral strain control mode. Besides, axial stress appear repeatedly cycle of falling and rising phenomenon. The volumetric strain-axial stress curve and axial strain–axial stress curves show similar behaviors. These feature don’t occur under axial strain control mode.

**Figure 4:**
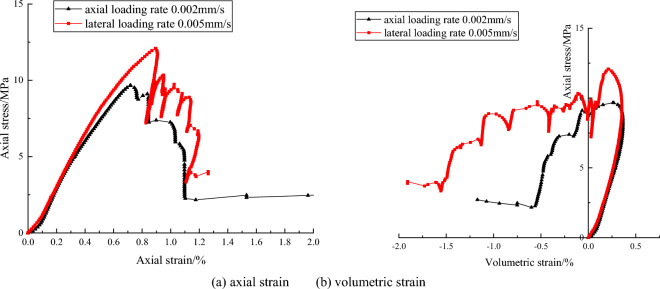
The complete stress and strain curve of mudstone under different loading rate and control mode.

The complete stress and strain curves of mudstone are shown in Fig. 4. The pre-peak stress and strain curves almost overlap very well in the beginning of loading under axial and lateral strain control mode. Axial loading rate is 0.002 mm/s, lateral loading rate is 0.005 mm/s. Due to difference of the loading rate, the peak strength under lateral control mode is about 13 MPa, the peak strength is greater than the peak strength of axial loading rate of 0.002 mm/s. What’s more, characteristic of post-peak stage has a big difference. The post-peak stress and strain curve also shows the feature of Class II behavior under lateral strain control mode. In other words, thought soft rock also shows the characteristic of Class II behavior at some appropriate lateral loading rate. Besides, axial stress also appear repeatedly cycle of falling and rising phenomenon. The volumetric strain-axial stress curve and axial strain–axial stress curve show similar behaviors. These feature don’t occur under axial strain control mode.

**Figure 5:**
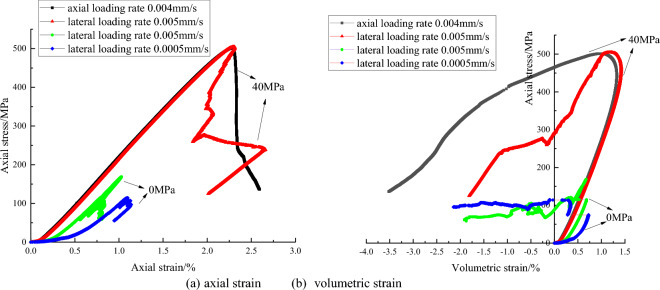
The complete stress and strain curve of granite under different loading rate and control mode.

The complete stress and strain curves of granite are shown in Fig. 5. Granite is a hard and brittle material. The failure process is violent in the uniaxial compression test under axial strain control mode. In order to protect the extensometer from damage, only the uniaxial compression tests under lateral strain control mode are conducted. Under lateral strain control mode, the stress and strain is similar in the pre-peak stage and stress increases with the axial strain. The peak strength under lateral loading rate of 0.005 mm/s is about 170 MPa. The stress and strain curve also shows the feature of Class II behavior in the post-peak stage. However, under lateral loading rate of 0.0005 mm/s, stress and strain curve show greater nonlinear feature in pre-peak stage and there are not an obvious peak strength like that of loading rate 0.005 mm/s. The possible reason is local unstable damage due to inhomogeneity of granite when axial stress approach the peak strength.

Under high confining pressure, the pre-peak stress and strain curves almost overlap very well, but there is a difference in the post-peak stage. The axial stress decreases and axial strain increases under axial strain control mode, showing the feature of Class I behavior. Both axial stress and axial strain decrease under lateral strain control mode, showing the feature of Class II behavior. It should be noted that the stress and strain curve don’t appear obvious repeatedly cycle of falling and rising phenomenon. The volumetric strain-axial stress curve and axial strain–axial stress curve show similar behaviors.

Based on the results of the complete stress and strain curve of four type rock samples, it can be seen that control mode has a great influence on the complete stress and strain curves. In pre-peak stage, stress and strain curves have same variation trend for four rocks, especially the stress and strain curves almost overlap in some situations. However, in post-peak stage, under axial strain control mode, four rock samples show the feature of Class I behavior. Under lateral strain control mode, four rock samples show the feature of Class II behavior or mixed Class I and Class II behavior, whether it is hard and brittle granite, moderate hard sandstone or soft mudstone.

Loading rate also influence the complete stress and strain curves. Under axial strain control mode, for red sandstone in uniaxial compression tests, the faster the axial loading rate is, the greater the peak strength is. Under lateral strain control mode, for gray sandstone in uniaxial compression test, with lateral loading rates decrease, post-peak stress and strain curves appear more and more obvious fluctuations in the post-peak stage and axial stress appear repeatedly cycle of falling and rising phenomenon. The main reason may be that the unstable cracks propagation in the samples. These unstable cracks induces the lateral expansion deformation when rock samples are suffered from the high axial stress. Under lateral strain control mode, the lateral deformation rate is constant, the rock sample is controlled to dilate uniformly. The larger lateral expansion deformation is restricted due to the servo feedback system. The suddenly increasing dilation rate of rock at the post-peak stage will lend to a sudden increase of the lateral strain rate. The servo-control system have to adjust the actuator to unload to reduce the increased lateral strain rate to keep the lateral strain rate constant, which will result in decrease of the axial stress or axial deviatoric stress. Axial strain will also be reduced synchronously. Therefore, the Class I or Class II types curve may occur and be shown in stress and strain curve. In pre-post stage, the axial stress is adjusted by working on the testing machine under lateral strain control mode. The elastic energy in the sample is released, axial stress decrease and the axial rebound deformation occur, so stress and strain curve show the characteristic of Class II behavior. With the internal stress distribution, the higher strength zone will bear more pressure. The axial stress increase locally. Once the pressure exceeds the bearing capacity of higher strength zone, this part of the rock will rupture again, the elastic strain energy release in the rock sample, rebound deformation occur. Both axial stress and axial strain decrease again. Rock failure is a process of repeated stress adjustment, so axial stress will be repeated cycle of falling and rising. However, due to the axial stress adjustment occur locally, so its value is less than the peak strength.

Besides, confining pressure also influence the complete stress and strain curves. Take the granite under confining pressure 40 MPa for example, under high confining pressure, the lateral dilation deformation is restricted, so peak strength is larger and stress redistribution can be better adjusted and rebond deformation reduced accordingly in post-peak stage.

### Strength characteristics

Stress thresholds is an important strength parameter during the deformation process of rocks under compression. Crack initiation stress $\sigma_{ci}$ is determined by crack volumetric strain method, crack damage stress $\sigma_{cd}$ is determined by reversal point stress value of total volumetric strain curve, and the peak stress $\sigma_{p}$ is the maximum stress value of complete stress–strain curve. The tests data are processed to obtain the crack initiation stress $\sigma_{ci}$ and corresponding strain, crack damage stress $\sigma_{cd}$ and corresponding strain, the peak stress $\sigma_{p}$ and corresponding strain. The results are shown in Table 3.

**Table 3:** Results of rock compression test.

Rock type	Axial loading rate	Lateral loading rate/mm/s	$\sigma_{{{\text{c}}i}}$/MPa	$\varepsilon_{ci}$/10^–3^	$\sigma_{cd}$/MPa	$\varepsilon_{cd}$/10^–3^	$\sigma_{p}$/MPa	$\varepsilon_{p}$/10^–3^
Gray sandstone	0.001	–	21.70	3.68	37.54	6.14	52.86	9.18
	0.001	0.0005	21.86	4.54	38.52	6.87	54.09	9.66
	0.001	0.0003	19.37	4.03	36.60	5.12	50.05	9.02
	0.001	0.0001	21.61	4.25	36.32	6.43	52.58	9.31
Red sandstone	0.001	–	32.49	4.96	41.73	5.89	63.05	8.30
	0.002	–	37.52	5.79	45.36	6.61	68.75	9.58
	0.001	0.001	36.64	4.70	42.92	5.58	60.01	7.93
Mudstone	0.002	–	6.18	4.11	8.24	5.72	9.65	7.15
	0.002	0.005	6.24	3.82	9.10	5.81	12.08	8.95
Granite	0.002	0.005	27.74	3.69	113.52	7.84	115.49	7.94
	0.002	0.0005	31.52	6.14	74.58	8.78	114.45	10.98
	0.004	–	345.73	15.17	460.91	20.35	501.28	22.76
	0.004	0.005	348.64	15.38	445.32	19.52	504.79	22.94

It can be seen from the Table 3, for gray sandstone, the crack initiation stress, crack damage stress and peak stress under axial control mode are basically the same as those under lateral control mode, indicating that the control mode has little influence on the stress thresholds before the peak. Compared with different lateral loading rates, the stress thresholds have also no significant change, indicating that the lateral loading rate has little influence on the strength property before the peak. In the post-peak stage, under axial control mode, peak strength rapidly decreases to a low value, while under lateral control mode, the post-peak strength decays slowly and there is no obvious residual strength value. With the decrease of lateral loading rate, the post-peak strengths have obvious rebound phenomenon, and the smaller the loading rate is, the larger the rebound amplitude is. For red sandstone, the peak strength increases with the increase of axial loading rate under axial control mode, but the crack initiation stress and crack damage stress change little. Compared with the lateral control mode, the pre-peak stress thresholds have no significant change, but the post-peak strength is different. The larger the axial rate is, the smaller the residual strength is, the lateral control has no obvious effect on the residual strength. The residual strength is about 19 MPa. For mudstone, rock strength is relatively low, and the deformation is mainly plastic deformation without obvious brittleness. Compared with axial control mode, the crack initiation stress, crack damage stress and peak stress are increased under the greater lateral control rate. In the post-peak stage, the residual strength is also greater than the strength under axial control mode. For granite, in uniaxial compression tests, its strength fluctuates greatly due to heterogeneity. Under high confining pressure, the pre-peak crack initiation stress, crack damage stress and peak stress are close to each other, indicating that high confining pressure can effectively limit the lateral deformation of rock and slow down the damage process. In the post-peak stage, there is a relatively constant residual strength under lateral control mode. But there is no significant residual strength under axial control mode. In summary, control mode has little influence on the stress thresholds and pre-peak strength property under the condition of the same speed, but has an effect on post-peak strength. Under lateral control mode, the post-peak strength may have repeated rebound, and its residual strength is more likely to have relatively stable and obvious residual strength, and the value is generally greater than that of axial control mode. The lateral control rate can make the rebound amplitude of post-peak rebound stress larger to some extent.

### Brittleness characteristic

Brittleness is an important mechanical index of rock, which is of great significance for rock mass evaluation and disaster prevention. Many brittleness index have been proposed to characterize rock brittleness behavior^21–23^. In order to study the brittleness characteristic of rock under different lateral loading rate, the results of gray sandstone in uniaxial compression tests are adopted. The brittleness index of gray sandstone under different lateral loading rates is calculated based on method of the literature^24^. The brittleness index is a method with clear physical meaning and monotonous and continuous relationship between calculation results and rock brittleness, based on the complete stress–strain curve of rock and the influence of pre-peak stress rising rate, post-peak stress falling rate and peak point strain. The calculation formulas are as follows:

1 $${\text{BI = }}B_{i1} B_{i2} B_{i3} ,$$

2 $$B_{i1} = \frac{{\sigma_{p} - \sigma_{ci} }}{{\varepsilon_{p} - \varepsilon_{ci} }},$$

3 $$B_{i2} = e^{{\frac{{10(\varepsilon_{p} - \varepsilon_{r} )}}{{\sigma_{p} - \sigma_{r} }}}} ,$$

4 $$B_{i3} = \frac{1}{{\varepsilon_{p} }}.$$

In the equation, ${\text{BI}}$ is the brittleness index of rock, $B_{i1}$, $B_{i2}$ and $B_{i3}$ are the pre-peak brittleness index, the post-peak brittleness index and the strain control coefficient respectively. $\sigma_{ci}$, $\sigma_{p}$, $\sigma_{r}$ are crack initial stress, peak stress, residual stress respectively. $\varepsilon_{ci}$, $\varepsilon_{p}$, $\varepsilon_{r}$ are crack initial strain, peak strain and residual strain respectively.

Due to the repeated rising and falling of the axial stress in post-peak stage under lateral control mode, the stress and strain corresponding to the end of the first drop section is calculated as the residual stress and strain here, and the calculation results are shown in Table 4. The brittleness index results show that brittleness index of granite is largest. Brittleness index of red sandstone is larger than that of gray sandstone. Mudstone is soft rock and the brittleness index is almost zero. Besides, the brittleness index is basically the same under different lateral control rates for gray sandstone, indicating that the lateral control rate has little significant effect on the brittleness index. But control mode has a significant effect on the brittleness index. The value of brittleness index under lateral control mode is larger than that of axial control mode for four rocks.

**Table 4:** Brittleness index results of rocks under different control mode and control rate.

Rock type	Axial loading rate	Lateral loading rate/mm/s	$\sigma_{{{\text{c}}i}}$/MPa	$\varepsilon_{ci}$/10^–3^	$\sigma_{p}$/MPa	$\varepsilon_{p}$/10^–3^	$\sigma_{r}$/MPa	$\varepsilon_{r}$/10^–3^	*B*_i1_	*B*_i2_	*B*_i3_	BI
Gray sandstone	0.001	–	21.70	3.68	52.86	9.18	9.73	9.72	5.67	0.88	0.11	0.54
	0.001	0.0005	21.86	4.54	54.09	9.66	31.51	8.74	6.29	1.50	0.10	0.98
	0.001	0.0003	19.37	4.03	50.05	9.02	34.64	8.23	6.15	1.67	0.11	1.14
	0.001	0.0001	21.61	4.25	52.58	9.31	33.81	8.32	6.12	1.69	0.11	1.11
Red sandstone	0.001	–	32.49	4.96	63.05	8.3	17.14	9.36	9.15	0.79	0.12	0.88
	0.002	–	37.52	5.79	68.75	9.58	1.9	10.1	8.24	0.93	0.10	0.80
	0.001	0.001	36.64	4.7	60.01	7.93	34.22	7.04	7.24	1.41	0.13	1.29
Mudstone	0.002	–	6.18	4.11	9.65	7.15	2.27	11.03	1.14	0.01	0.14	0.001
	0.002	0.005	6.24	3.82	12.08	8.95	7.22	8.27	1.14	4.05	0.11	0.52
Granite	0.002	0.005	27.74	3.69	115.49	7.94	67.5	6.53	20.65	1.34	0.13	3.49
	0.002	0.0005	31.52	6.14	114.45	10.98	55.46	9.51	17.13	1.28	0.09	2.00
	0.004	–	345.73	15.17	501.28	22.76	136.2	25.91	20.49	0.92	0.04	0.83
	0.004	0.005	348.64	15.38	504.79	22.94	259.65	18.36	20.65	1.21	0.04	1.09

### Failure modes

**Figure 6:**
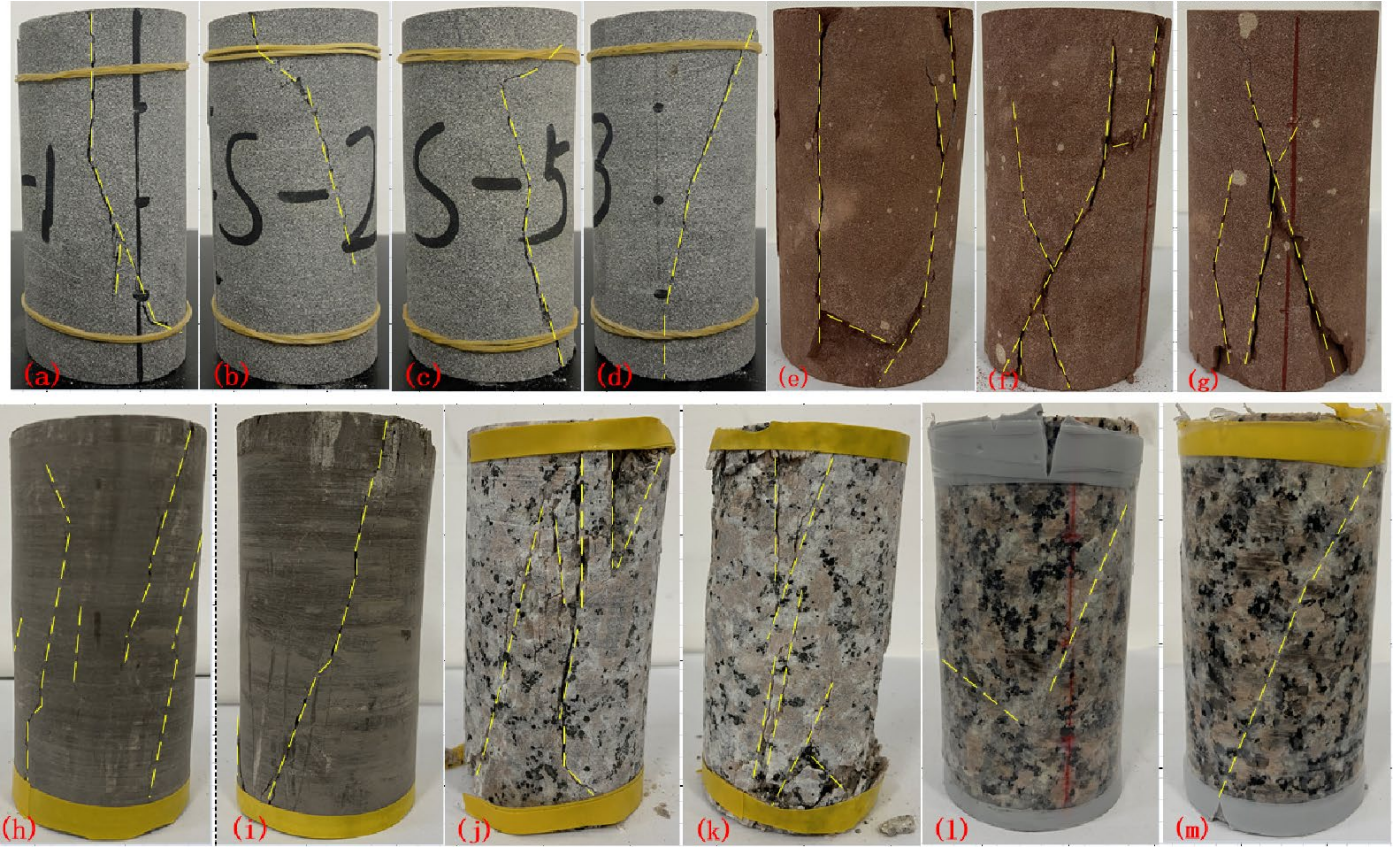
Failure mode of rock samples. Gray sandstone (**a**) axial loading rate 0.001 mm/s, (**b**) lateral loading rate 0.0005 mm/s, (**c**) lateral loading rate 0.0003 mm/s, (**d**) lateral loading rate 0.0001 mm/s. Red sandstone (**e**) axial loading rate 0.001 mm/s, (**f**) axial loading rate 0.002 mm/s, (**g**) lateral loading rate 0.001 mm/s. Mudstone (**h**) axial loading rate 0.002 mm/s, (**i**) lateral loading rate 0.005 mm/s. Granite (**j**) lateral loading rate 0.005 mm/s, (**k**) lateral loading rate 0.0005 mm/s, (**l**) axial loading rate 0.004 mm/s, (**m**) lateral loading rate 0.005 mm/s.

The photographs of rock samples after failure are shown in Fig. 6. From the perspective of failure mode, the failure mode is splitting and shear complicated failure modes. Figure 6a–d is photographs of gray sandstone. Gray sandstone samples under axial strain control mode show a nearly vertical splitting crack and shear crack combined failure. But samples under lateral strain control mode mainly show shear failure. As the lateral loading rate decreases, the shear failure becomes more obvious and a complete shear crack is formed. This indicates that the failure process under axial control mode is more severe, while under lateral strain control mode is much slow and stable. With the decrease of lateral loading rate, the failure process will be more stable and slower, and gradually transition from splitting and shear complicated failure to completely shear failure. Figure 6e–g is photographs of red sandstone. Red sandstone mainly suffers from vertical splitting failure under axial strain loading. The failure process becomes more intense with the increase of axial loading rate. That is to say, the greater the axial loading rate, the more serious the failure process is. Under lateral strain loading, the failure mode is mainly shear failure, and the failure process is relatively stable. Figure 6h,i is photographs of mudstone. The failure of mudstone is mainly shear failure with multiple shear slip traces under axial strain loading, while failure mode is mainly show a shear crack thought the rock sample under lateral strain loading. Figure 6j–m is photographs of granite after failure. The failure mode of granite is splitting failure under lateral strain loading. Compared with other rock samples, the failure process of granite is more seriously and there are obvious falling blocks. Under the high confining pressure of 40 MPa, the failure mode changes to shear failure. By comparing the failure mode of axial and lateral control mode under high confining pressure, the shear plane under lateral strain loading is more complete. High confining pressure can decrease the degree of damage and make the integrity of samples better.

According to the above analysis of the failure modes of different lithologies, the failure process of rock samples under lateral strain control mode is slower and more stable than that under axial strain control. The failure mode of rock samples under axial control is mainly splitting failure, while that under lateral strain control is gradually changed to shear failure. The smaller the lateral loading control rate is, the more obvious the shear failure characteristics is. Besides, failure modes have a relationship in with the brittleness index. From the Table 4, brittleness index of granite is largest, and failure mode of granite is splitting failure under axial loading condition. Some fragments fell away from the granite sample can be observed. The brittleness index of red sandstone is greater than that of gray stone under axial loading mode, so failure mode of red sandstone vertical splitting failure, but gray sandstone is vertical splitting crack and shear crack combined failure. Some fragments fell away from the red sandstone sample can be also observed. But the integrity of gray sandstone is better. Mudstone is a soft rock, brittleness index is almost zero, failure mode is shear failure with multiple shear slip traces.

## Mechanism analysis and discussion

**Figure 7:**
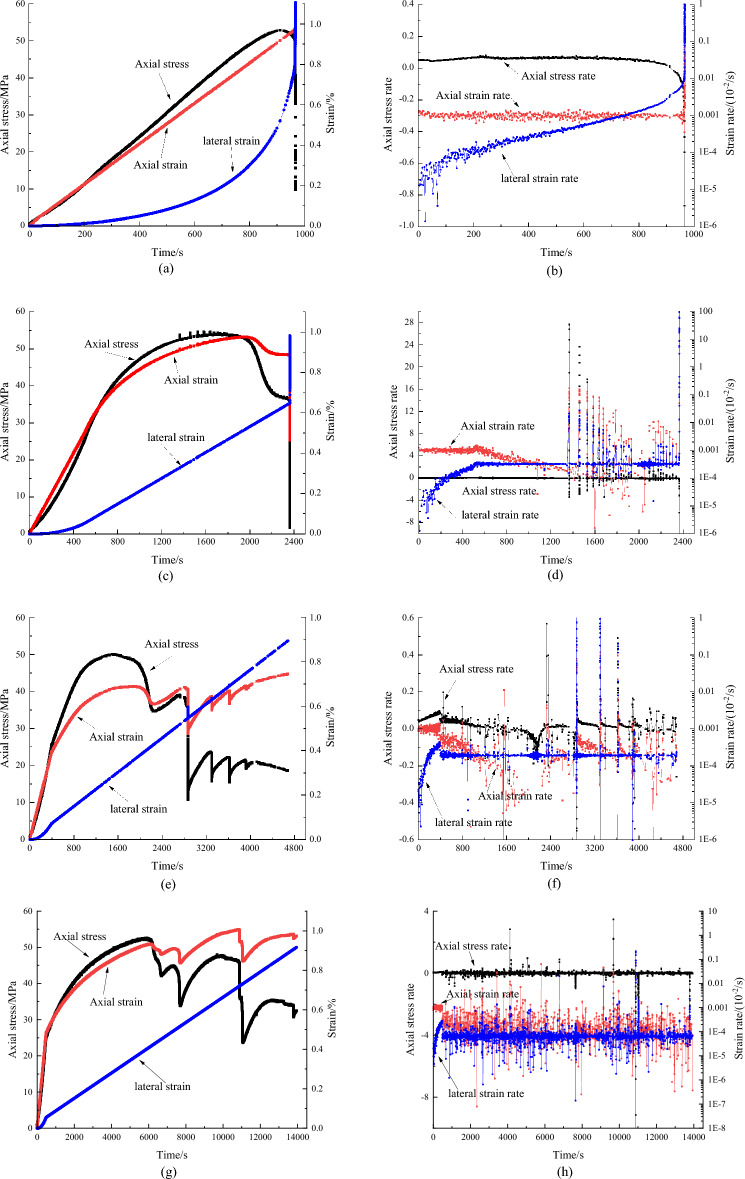
Variation of axial stress, axial strain, lateral strain, axial stress rate, axial strain rate and lateral strain rate for gray sandstone under different control mode and loading rate. (**a,b**) is axial strain control mode and axial loading rate is 0.001 mm/s; (**c,d**) is lateral control mode and lateral loading rate is 0.0005 mm/s; (**e,f**) is lateral control mode and lateral loading rate is 0.0003 mm/s; (**g,h**) is lateral control mode and lateral loading rate is 0.0001 mm/s.

In order to better understand the influence of control mode and loading rate on the complete stress and strain curves, the variations of axial stress, axial strain, lateral strain, axial stress rate, axial strain rate and lateral strain rate with time of gray sandstone are shown in Fig. 7. Figure 7a,b are the results of axial strain control mode and loading rate is 0.001 mm/s. Axial strain rate is a constant value of 1 × e^−5^, and axial stress increase with time at first, and then keep almost constant until 880 s. When axial stress approaches the peak stress, the value decrease gradually with time and after peak the value decrease rapidly. Lateral strain increases nonlinearly and the lateral strain rate increases gradually. When axial stress approaches the peak stress, the strain rate is faster, and after peak the strain rate increase rapidly.

Figure 7c–h are results of lateral strain control mode. Figure 7c,d show the results of lateral strain loading rate of 0.0005 mm/s. It can be seen from the figures, at the initial loading stage, due to axial strain control, its behavior is similar to the Fig. 7a,b. When the axial stress increases to 25 MPa, the control mode is switched from axial strain control mode to lateral strain control mode, axial stress, axial strain and lateral strain show different characteristic. The axial stress increases at a relatively constant rate. After 1300 s, the axial stress beyond the crack damage stress, the axial stress increase suddenly locally due to rock damage (crack develop and propagate), and axial stress rate shows impulsive phenomenon. The axial strain changes from linear increase to nonlinear increase after the change of control mode, and axial strain rate decreases gradually. Axial strain rate also shows the impulsive phenomenon due to local damage. Lateral strain changes from nonlinear increase to linear increase after the change of control mode, and lateral strain rate keeps a constant value. Lateral strain rate also shows the impulsive phenomenon due to local damage. After peak stress is reached, axial stress and axial strain decrease simultaneously. The stress and strain curve shows the characteristic of class II behavior (red line in Fig. 2a). Axial strain occurs rebound and the direction of axial stress and axial strain is opposite, so the elastic energy in the sample is released and rock sample is doing work to the loading system.

Figure 7e–h show the results of lateral strain loading rate of 0.0003 mm/s and 0.0001 mm/s respectively. The general law is the same as that of Fig. 7c,d. But lateral loading rate is different, there are some new characteristic. The smaller the lateral loading rate is, the slower the rock samples dilates, the longer the time of controllable and stable is. At loading rate is 0.0005 mm/s, the time is about 2380 s, while the time reach to 4700 s and 13000 s at rate of 0.0003 mm/s and 0.0001 mm/s and rock samples still are controllable. In addition, In the case of 0.0005 mm/s, the lateral loading rate changes from nonlinear to constant without abrupt change. While in the case of 0.0003 mm/s and 0.0001 mm/s, the lateral strain rate increases nonlinearly to a certain value and then sudden drops to a constant value, indicating that the lateral control rate at this time is inconsistent with the lateral expansion rate of the rock sample itself. The lateral control rate is greater than the lateral expansion rate of the rock sample, which makes the servo-feed system decrease axial stress and strain to reduce the lateral rate in order to makes the lateral deformation controllable. By contrast, in bigger lateral strain rate tests or axial strain control mode tests, the failure process of rock is instantaneous and uncontrollable. The reason for the fluctuation in Fig. 7h is that the lateral expansion rate is often greater than the lateral pre-defined control rate, causing the servo control system to adjust the process repeatedly.

Through the above analysis, we also further verified the influence of the control mode and loading rate on the complete stress and strain curve. The change of the control mode has influence on the pre-peak, mainly reflected in the longer time required to reach the peak, but has little influence on the peak strength, while it has a greater impact on both the time and deformation after peak, making the post-peak deformation stable and controllable, and show the feature of Class II behavior. The main reason is the reduction of axial force caused by the servo system due to the difference between the lateral expansion rate of rock and the lateral control rate. Class II stress–strain curves is a phenomenon caused by actuator unloading by the servo-control system, which has been removed by some scholars^9,10^. Here through our research, we found that different lateral control rates make a difference to post-peak stress and strain curve and laws.

In addition to the control mode and loading rate, we have also carried out comparative tests on different lithology samples, such as hard and brittle granite, moderate strength gray sandstone and red sandstone and lower strength mudstone. Through the analysis of the preliminary test results, four type of rock show the characteristic of Class II behavior, indicating that the formation of Class II curve can be caused by the control mode and appropriate loading rate, and has little relationship with the lithology. In other words, even for soft mudstone, the phenomenon of Class II curves will appear under appropriate lateral loading rate. The similar results of mixed Class I and Class II post-peak curves of mortar under lateral strain control mode have also been observed in recent literature^11^. In the case of tri-axial compression test, the brittleness of the post-peak curve of granite was weakened and the severity of failure was reduced due to high confining pressure under axial strain control mode. Under lateral strain control mode, the mechanical behavior appear more stable and controlled. On the one hand, lateral control mode makes the lateral dilatation slower, on the other hand, confining pressure can also limit lateral deformation. Both comprehensive result is more noticeable. Local rock fracture is significantly reduced. Although the strength has fluctuates, its adjustment range is significantly improved compared with that of the uniaxial compression test. The failure mode also changes from split failure to shear failure.

## Conclusion

1. Control mode has a great influence on the complete stress and strain curves. In pre-peak stage, stress and strain curves have same variation trend for four rocks, especially the stress and strain curves almost overlap in some situations. However, in post-peak stage, under axial strain control mode, four rock samples show the feature of Class I behavior. Under lateral strain control mode, four rock samples show the feature of Class II behavior or mixed Class I and Class II behavior.
2. Control mode has little influence on the stress thresholds and pre-peak strength property under the condition of the same speed, but has an effect on post-peak strength. Under lateral control mode, with lateral loading rates decrease, post-peak stress and strain curves appear more and more obvious fluctuations in the post-peak stage and axial stress appear repeatedly cycle of falling and rising phenomenon. Under high confining pressure, the lateral dilation deformation is restricted, so peak strength is larger and stress redistribution can be better adjusted and rebond deformation reduced accordingly in post-peak stage.
3. Lateral control rate has little significant effect on the brittleness index, but the value of brittleness index under lateral control mode is larger than that of axial control mode. The failure mode of rock samples under axial control is mainly splitting failure, while that under lateral strain control is gradually changed to shear failure. The smaller the lateral loading control rate is, the more obvious the shear failure characteristics is.

## Data Availability

All data used during the current study available from the corresponding author on reasonable request.
